# Emotion Recognition in Low-Spatial Frequencies Is Partly Preserved following Traumatic Brain Injury

**DOI:** 10.1155/2019/9562935

**Published:** 2019-01-28

**Authors:** Alessia Celeghin, Valentina Galetto, Marco Tamietto, Marina Zettin

**Affiliations:** ^1^Department of Psychology, University of Torino, 10123 Torino, Italy; ^2^Centre Hospitalier Universitaire Caremeau, Nîmes, France; ^3^Centro Puzzle, 10137 Torino, Italy; ^4^Department of Medical and Clinical Psychology, Tilburg University, 5000LE Tilburg, Netherlands; ^5^Netherlands Institute for Advances Study in the Humanities and Social Sciences (NIAS), Royal Netherlands Academy of Arts and Sciences (KNAW), 1001 EW Amsterdam, Netherlands

## Abstract

After a Traumatic Brain Injury (TBI), emotion recognition is typically impaired. This is commonly attributed to widespread multifocal damage in cortical areas involved in emotion processing as well as to Diffuse Axonal Injury (DAI). However, current models suggest that emotional recognition is subserved by a distributed network cantered on the amygdala, which involves both cortical and subcortical structures. While the cortical system is preferentially tuned to process high spatial frequencies, the subcortical networks are more sensitive to low-spatial frequencies. The aim of this study was to evaluate whether emotion perception from low-spatial frequencies underpinning the subcortical system is relatively preserved in TBI patients. We tested a group of 14 subjects with severe TBI and 20 matched healthy controls. Each participant was asked to recognize the emotion expressed by each stimulus that consisted of happy and fearful faces, filtered for their low and high spatial frequencies components. Results in TBI patients' performances showed that low-spatial frequency expressions were recognized with higher accuracy and faster reaction times when compared to high spatial frequency stimuli. On the contrary, healthy controls did not show any effect in the two conditions, neither for response accuracy nor for reaction times. The outcomes of this study indicate that emotion perception from low-spatial frequencies is relatively preserved in TBI, thereby suggesting spare of functioning in the subcortical system in mediating emotion recognition.

## 1. Introduction

Traumatic brain injury (TBI) is a frequent cause of disability and leads to notable and aversive effects on different social and cognitive skills [1, 2]. Although changes in emotional behaviour of patients with TBI have long been noticed, their experimental investigation, as well as the connection with every-day postinjury social abilities, has come under scrutiny only in recent years [1, 2]. In fact, studies on the clinical outcomes of TBI reported clear difficulties in multiple neuropsychological functions, with the greatest limitations in interpersonal communication abilities and emotional health, which often manifest in reduced employment, restricted family and social relations, and overall reduction in quality life [3]. Emotion perception and recognition is a critical component of, and a necessary antecedent for, social competences. In fact, facial expressions are salient cues that ostensibly communicate one's feelings [4]. Persons with TBI have significant deficits in recognizing nonverbal cues of affect, especially from facial expressions, and these impairments have been demonstrated primarily through tests requiring patients to label or match facial expressions. Moreover, numerous studies have shown that people who are impaired at reading social cues also experience poor social skills, emotional liability or disinhibition, reduced feelings or blunting affects [5–7].

Yet relatively little is known about the specific profile of functional alterations in TBI which underlie deficits in emotion perception from faces [3, 4, 8–13]. For example, the face coveys simultaneously different information about fine details or global orientation and proportions, but there is currently no investigation on whether these different aspects are selectively damaged or relatively preserved following TBI. Neuroimaging studies have revealed that the neural underpinnings of emotion perception from faces involve a complex network of cortical and subcortical structures, such as the fusiform gyrus, the orbitofrontal cortex, the insula, and the amygdala [14–17]. Moreover, neuropsychological studies in patients with focal brain damage reported double dissociations amid different aspects of social processing from facial information, such as identity, familiarity, or emotional expressions, thereby providing causal evidence that multiple features and different processing strategies engage partly segregated neural substrates [15–19].

Notably, a classical model of emotion perception holds that the amygdala, a key subcortical structure for encoding emotional signals, receives two parallel sources of visual information. A direct subcortical input from the superior colliculus and pulvinar enables fast but coarse processing of affective information and a slower but more detailed and fine-grained cortical input involving long-range connectional tracts from the ventral visual pathway [20, 21]. Neurophysiological, neuroimaging, and behavioural studies portend that these two processing streams to the amygdala have different roles in emotion perception from faces. The former subcortical pathway is phylogenetically ancient, draws primarily on magnocellular input, and is selectively tuned to lower spatial frequencies (LSF) (2-8 cycles/face), which convey global configurational information sufficient to enable fast and coarse perception of emotional expressions. The latter cortical pathway is more recent phylogenetically, receives substantial parvocellular input, and responds chiefly to high spatial frequencies (HSF) (8-16 cycles/face), which convey slow and fine-grained information important for detailed analysis of facial traits. Therefore, it is possible to examine the selective contribution of these two visual pathways by exploiting their distinct sensitivities to different ranges of spatial frequencies.

No prior study has yet investigated possible differences of emotion perception in patients with TBI as a function of spatial frequencies in the stimuli. This is surprising due to both theoretical and clinical relevance of such inquiry. Concerning theoretical relevance, TBI offers an interesting “experimental model” to disentangle the role of the cortical and subcortical processing pathways in emotion recognition. In fact, emotion recognition is damaged mostly because cortical areas pivotal to affective processing, such as the orbitofrontal cortex, parietal or temporal areas along the lateral and orbital surface of the brain are particularly vulnerable to TBI [3, 8, 10, 11, 22–26]. These areas are adjacent to points of impact in the skull cavity, and multifocal lesions at these sites often follow from brain jolting, rapid head acceleration and rotation [4, 10, 24, 25, 27]. Moreover, the same biomechanical events cause diffuse axonal injury (DAI) that results in tearing off and fragmentation of white matter tracts, particularly long-distance association pathways running along the anterior-posterior axis, such as those involved in providing cortical input to the amygdala [13]. Conversely, subcortical structures such as the superior colliculus, the pulvinar and the amygdala itself are relatively less liable to TBI. Their anatomical location, buried deeply into the brain, lays closer to the geometrical centre of gravity than cortical areas on the lateral or orbital surface. Furthermore, short-range connections in the limbic system, such as the fornix and the stria terminalis, or those reported to connect the superior colliculus with the amygdala through the lateral pulvinar [28], are less susceptible to DAI. Concerning clinical relevance, deficits of emotion recognition in TBI patients can be considered as an early marker of behavioural problems and lack on insight. Detection of emotion recognition deficits, as well as the possibility to identify potentially preserved subcomponents of this processing, may allow early treatment of these problems, thereby implementing either restorative or compensatory rehabilitation strategies.

In the present study, we decomposed facial expressions into their LSF and HSF components to address the specific functional properties of partly segregated pathway to the amygdala. To this end, we aimed at delving into the nature of emotion recognition deficits in TBI patients and we predicted to reveal sparing of functions thus far undescribed. Specifically, our contention was that TBI patients should present with higher accuracy and slower reaction times in recognizing facial expressions displaying only LSF than HSF components, owing to the fact that subcortical pathways for emotion perception are relatively less compromised than cortical pathways.

## 2. Materials and Methods

### 2.1. Participants

Fourteen subjects with TBI (4 females) and 20 healthy controls (HC; 7 females) were enrolled in this study. TBI subjects were recruited at Centro Puzzle, a local rehabilitation centre for patients with severe brain injury. All the patients presented with severe TBI according to positive neuroimaging findings during acute-care hospitalization and based on their score at the Glasgow Coma Scale (GCS; [29]), which is typically used as a clinical indicator of injury severity in TBI. The GCS of all the patients included in the study was equal to or less than 8. Out of 14 TBI patients, 9 suffered from DAI as the consequence of an accident occurred to the driver or passenger of a moving vehicle, while 2 patients as pedestrian struck by a moving vehicle. At the time of the study, all the patients were in the chronic phase of their injury and under neurorehabilitation treatment. Exclusion criteria were more than one TBI, neurological conditions other than TBI (e.g., strokes, tumour, seizures, and neurodegenerative disorders), psychiatric conditions (e.g., major depression, bipolar disorder), and substance abuse before or after injury.

**Figure 1:**
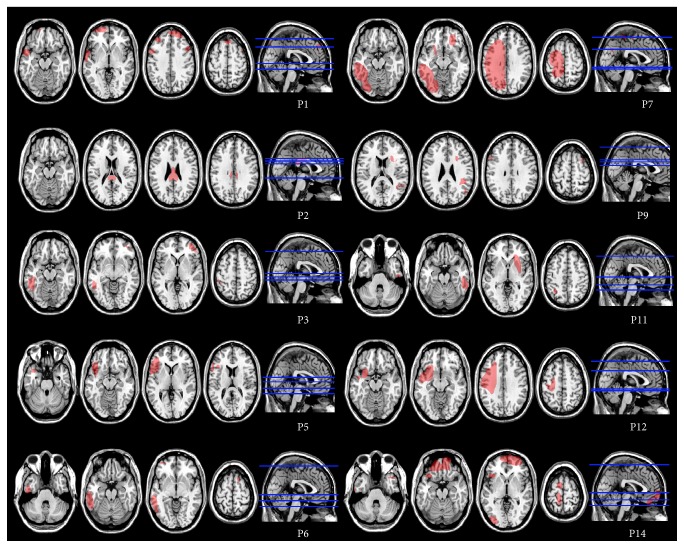
Lesion reconstruction images from MRI or CT scans projected onto the normalized MNI template for the 10 TBI patients with detectable focal lesions.

**Figure 2:**
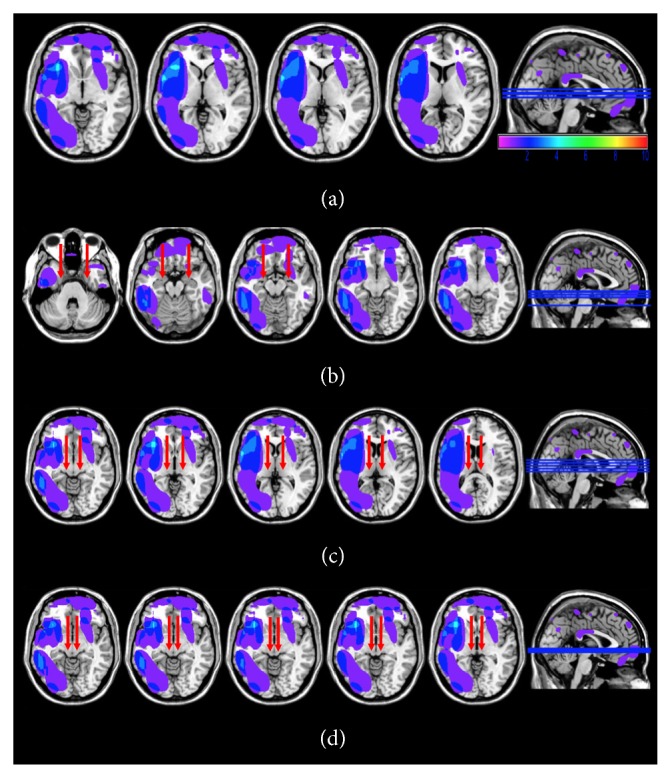
Location and overlap of brain lesions projected onto axial slices of the standard MNI brain (a). The arrows indicate the location of the amygdala (b), pulvinar (c), and superior colliculus (d) and document that these structures were spared in all patients with focal lesions.

Ten patients had detectable signs of focal brain lesions, as assessed by magnetic resonance imaging (MRI) or computerized tomography (CT) scan. Brain lesions were mapped using MRIcro [30, 31], based on the most recent clinical CT or MRI available. Lesions were traced onto the T1-weighted MRI templates from the Montreal Neurological Institute that matched Talairach space, with the exception of P4 and P10, whose MRI scans were not available (Figure 1). As shown in lesions overlaps, subcortical structures involved in encoding facial expressions based on low-spatial frequencies, such as the superior colliculus, the pulvinar, and the amygdala, did not show detectable sign of damage (Figure 2). Conversely, lesions were appreciable in multiple cortical regions of the frontal, temporal, and parietal lobes, predominantly on the lateral surface, and in the orbital surface of the frontal lobes. Areas of maximum overlap were centred on the ventro-lateral prefrontal cortex, insular cortex, and to a lesser extent in temporal areas along ventral visual stream in the middle and inferior temporal gyrus.

Patients' performance was compared with that of twenty healthy controls recruited from the local community and matched for age, gender, and education (Table 1). Inclusion criteria were (1) vision acuity within the normal range; (2) no reported history of psychiatric illness; (3) no preexisting neurological conditions, such as stroke, dementia, or head injury; and (4) no history of substance abuse within the past 12 months. Written informed consent was obtained from all the participants after detailed explanation of the study. Approval for the study had previously been obtained from the local ethic committee.

### 2.2. Neuropsychological Assessment

All TBI participants underwent neuropsychological assessment to evaluate the cognitive functions and rule out degenerative disorders. General cognitive impairments were tested with the Mini Mental State Examination questionnaire (MMSE, [32]). Emotion recognition from facial expressions was assessed with the Facial Expressions of Emotion: Stimuli and Tests (FEEST), specifically the subset composing the Ekman 60 Faces Test. It consists of 60 black-and-white pictures displaying 10 facial expressions for each of the six basic emotions (i.e., happiness, sadness, surprise, fear, anger, or disgust [33, 34]). Stimuli are presented for 5 sec, after which the subject has to choose which emotional label best describes the expression shown in the face. The total score ranges from 0–60, whereas the score for each emotion ranges from 0–10 (Table 2). The test has significant split-half reliability for the global score as well as for scores associated with individual emotions. Likewise, validity was excellent, as reported by the authors, with recognition rate of two different normative groups exceeding a correlation score of 0.8. Finally, the test has proven to be sensitive to emotion deficits in clinical groups, including TBI patients [12].

### 2.3. Stimuli

**Figure 3:**
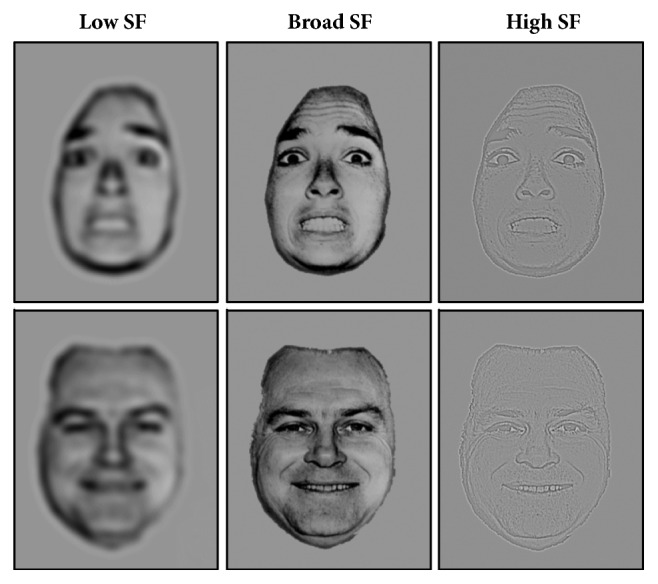
Example of happy and fearful faces unfiltered (BSF), filtered by low-spatial frequencies (LSF) and by high spatial frequencies (HSF).

Stimuli were 8 black-and-white photographs of emotional faces taken from the Ekman set [33], of which 4 expressing fear and 4 expressing happiness. All faces excluded most of the hair and nonfacial contours. In keeping with previous psychophysical and neuroimaging studies [35–37], the spatial frequency content in the original picture (broad-band spatial frequency, BSF) was manipulated using a 2D isotropic Butterworth filter. LSF images were created applying a low-pass cut-off of 6 cycles/image, whereas the LSF stimuli were generated applying a high-pass cut-off of 24 cycles/image width (see Figure 3 for an example). Numerical filtering using MATLAB [38] was applied in the spatial frequency domain and the result was then Fourier transformed into the spatial domain. This spatial frequency manipulation was tailored to engage selectively the magnocellular subcortical pathway for LSF images and the parvocellular cortical pathway for HSF images. Therefore, we obtained a set of 12 stimuli for each of the two emotions: 4 LSF, 4 HSF, and 4 BSF.

### 2.4. Experimental Task

Participants were seated in a dimly lit room in front of a 15” computer screen with a resolution of 1024 x 768 pixels, at a viewing distance of 30 cm. The experiment consisted of one practice block and 4 main blocks of 30 randomized trials each, for a total of 120 trials; 40 LSF, 40 HSF, 40 BSF stimuli were equally distributed between happiness and fear. Each trial began with a fixation cross over a grey background (RGB: 160, 160, 160) followed by the central presentation of a face stimulus for 2000 ms. Participants were asked to discriminate between the two emotions (Fear vs. Happiness) as soon as possible by pressing two response buttons of the keyboard. Intertrial interval was self-paced. Recognition accuracy and reaction times (RTs) were recorded from stimulus onset.

### 2.5. Data Analysis

We first compare the groups on sociodemographic variables using a multivariate analysis of variance (ANOVA). To evaluate the neuropsychological condition and to assess overall facial emotion recognition, we compared the results obtained from each patient with a cut-off score. In order to evaluate the emotion recognition of fear and happiness filtered by the different spatial frequencies, we compared mean correct responses and reaction times, both in healthy control group and TBI patients by means of repeated-measures ANOVAs.

## 3. Results

### 3.1. Emotion Recognition at the FEEST

Mental deterioration can be ruled out in all the TBI participants, as demonstrated by the fact that their performance at the MMSE was above the cut-off value (≥ 25/30). Table 2 shows the score (correct recognition) of each TBI patient along with the means and SD at the FEEST total score and FEEST subscores for different emotions. Comparisons with normal population were derived from test validation, which included 227 individuals aged 20-70 years with IQ of 90 and above [39]. Mean correct recognition rates and cut-off scores defining the border between normal range and impaired performance (p = .05) for the population subgroups based on age were compared against each patient for total score and subscores in different emotions. Results are compatible with those previously reported in other studies examining emotion recognition in patients with moderate and severe TBI and show that the patients tested in this study had significant difficulties in recognizing basic emotions. In fact, all patients were below the normal cut-off total score.

### 3.2. Accuracy

**Figure 4:**
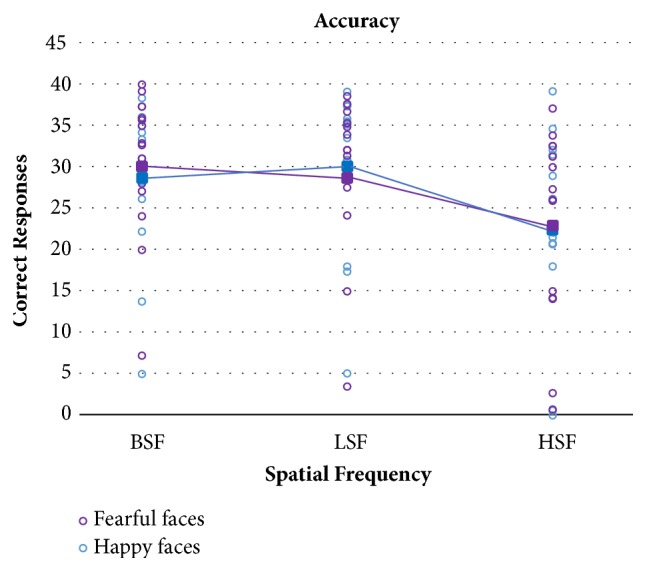
TBI participants' mean accuracy (filled squares) and single patients' performance (empty circles) in the three spatial frequency conditions for happy and fearful faces separately.

**Figure 5:**
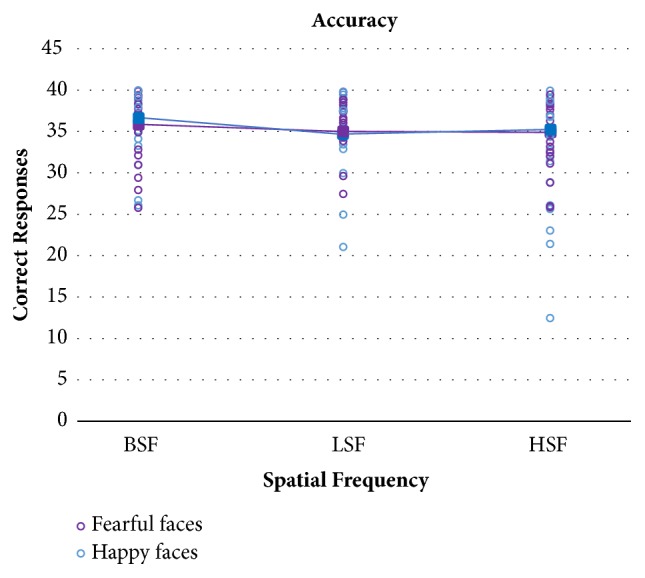
Healthy controls' mean accuracy (filled squares) and single participants' performance (empty circles) in the three spatial frequency conditions for happy and fearful faces separately.

Figure 4 displays mean accuracy and single performance in TBI patients as a function of fearful and happy facial expressions and spatial frequencies, while Figure 5 shows the same data in the control group.

Recognition accuracy was analysed with a mixed 2x2x3 ANOVA with the between-subjects factor Group (TBI patients vs. Controls) and the within-subjects factors Emotions (Fear vs. Happy) and Spatial Frequencies (BSF, LSF, HSF). Unsurprisingly, the main effect of Group turned out to be significant, with patients performing significantly less accurately in emotion recognition than matched controls (F(1,32)=14.02; p=0.0007). The main within-subject effect of emotions was not significant (F(1,32)=0.005; p=0.94), nor any of the interactions with this factor (p≥0.32). The main effect of spatial frequencies was statistically significant (F(2,64)=26.8; p<0.0001). Crucially, the interaction between Group and Spatial Frequencies was significant, indicating that the frequency manipulation was selectively effective in the TBI group (F(2,64)=17.5; p<0.0001). Post hoc Bonferroni comparisons on this interaction revealed indeed that accuracy in recognizing HSF emotions was significantly reduced compared to recognition of the same emotions in BSF or LSF, but only in the TBI group (p≤0.0001). No other post hoc comparison or interactions were significant (p≥0.5).

### 3.3. Reaction Times

**Figure 6:**
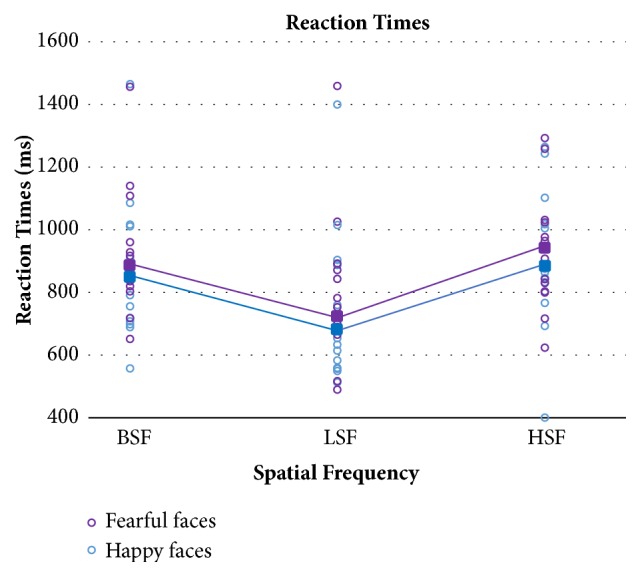
TBI participants' mean reaction times (filled squares) and single patients' performance (empty circles) in the three spatial frequency conditions for happy and fearful faces separately.

**Figure 7:**
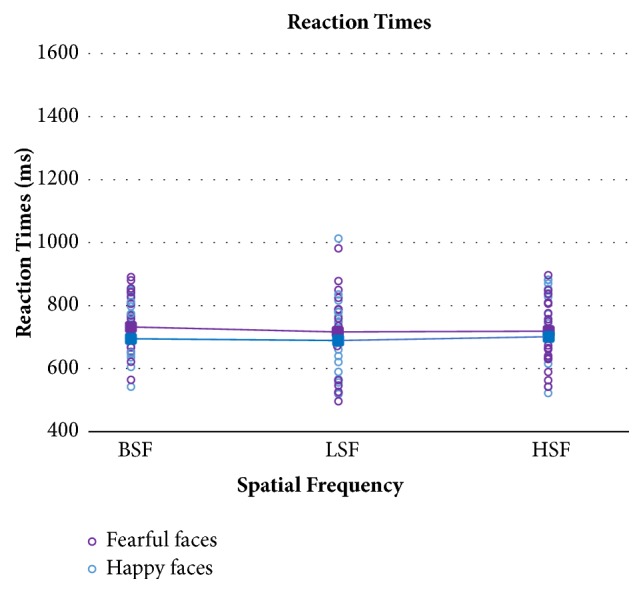
Healthy controls' mean reaction times (filled squares) and single participants' performance (empty circles) in the three spatial frequency conditions for happy and fearful faces separately.

Figure 6 displays mean RTs and single performance in TBI patients as a function of fearful and happy facial expressions and spatial frequencies, while Figure 7 shows the same data in the control group.

The same mixed ANOVA model, with the same factors and levels described in the accuracy analysis, was also applied to RTs. Again, TBI patients responded more slowly than controls, as shown by the significant main effect of Group (F(1,32)=7.49; p=0.01). The main within-subject effect of emotions was significant, showing faster RTs to happy than fearful expressions in TBI patients as well as healthy controls (F(1,32)=6.15; p=0.019). The main effect of spatial frequencies was statistically significant (F(2,64)=10.28; p=0.0001), as well as the Group x Spatial Frequencies interaction (F(2,64)=7.63; p=0.001). Post hoc tests on the interaction showed that in TBI patients RTs were faster for the recognition on LSF compared to both HSF and BSF.

## 4. Discussion

A signature deficit of TBI concerns breakdown in emotional, social, and interpersonal skills that impact on long-term recovery [1, 2]. However, relatively little is known about the underlying neuropsychological mechanisms, as well as on the unique mixture of impaired and preserved basic functions that characterize the nature of affective disorders in TBI. This is not only an important endeavour of scientific inquiry, but also a key step to envisage rehabilitative interventions that capitalize on spared subcomponents within different functional domains [3, 4, 8–13].

To this end, we investigated emotion recognition in a group of 14 TBI subjects exploiting the unique neurophysiological properties concerning the distinct contribution of the subcortical-magnocellular and cortical-parvocellular system to emotion perception. In fact, by tailoring stimuli that primarily engage just one of the two pathways at a time, we were able to reveal in more detail the nature of emotion recognition deficits in TBI. In line with prior studies, we found that emotion recognition was impaired in TBI patients compared to healthy population. This emerged both from the neuropsychological assessment with FEEST and from the results of the experimental task, where patients were overall slower and less accurate than healthy controls. In the former task, the patients had to discriminate among black-and-white faces of happiness, fear, anger, sadness, disgust, and surprise. Results indicate a generalized impairment in recognizing emotions, which is particularly exacerbated for negative expressions. The number of errors increased for the negative emotions such as fear, anger, surprise, and disgust, while a more accurate recognition was observed for happy expression. Both these results are in line with previous works [40–43] and advocate different possible explanations. Happiness is the only basic emotion with positive valence and hence may be the easiest to recognize, whereas multiple alternatives are possible among expression of negative valence [41]. Alternatively, distinctive and unique physical features characterize happy expressions with respect to negative emotions, which in turn share some visual features such as exposure of eye whites or frowning which make them more difficult to distinguish.

Notably, the accuracy difference between fearful and happy expressions was present in the FEEST clinical assessment but not in the experimental task. In this case, participants were asked to recognize emotions in BSF, LSF, and HSF in a 2-alternatives forced-choice task (2AFC). Therefore, emotion discrimination in the experimental task was contextualized within an easier set-up (e.g., 2AFC task vs. 6AFC), which may have overshadowed subtle differences in emotion discrimination that can possibly emerge only when more options are given. An additional novelty of the design was to combine measures of accuracy and reaction times, as previous studies on emotion recognition primarily focused only on accuracy. This was possibly due to the fact that measuring RTs in TBI patients is often problematic because responses are often delayed, thereby affecting reliability. Therefore, a note of caution should be considered when interpreting RTs data. However, we have tried to circumvent this difficulty by adopting a simplified task that required patients to choose only between two alternative expressions.

The main thrust of the present study is to show that spatial frequency manipulation affected emotion recognition in TBI patients in terms of both accuracy and response times. Concerning accuracy, while emotion recognition was equivalent in BSF and LSF, it was significantly impaired when faces were filtered so as to display only HSF components. As for RTs, recognition was faster for LSF expressions with respect to both HSF and BSF stimuli. This convergence of accuracy and reaction times data rules out any explanation in terms of speed-accuracy trade-off.

To our knowledge, the present findings provide the first empirical support to the proposition that emotion recognition in TBI is relatively preserved for the visual components to which the subcortical pathway to the amygdala is selectively sensitive (i.e., LSF). This is in keeping with prior studies in healthy and brain damaged patients showing that LSF expressions selectively engage subcortical pathway to the amygdala [28, 37, 44–55]. It is also in keeping with evidence that multifocal lesions and DAI in TBI patients impact primarily cortical areas of the frontal and temporal lobe known to be critical for emotion perception as well as in the association projections connecting these areas [56]. This uneven distribution of the brain lesions following TBI has also been directly confirmed in our sample by the assessment of the lesion distributions. Better proficiency in recognizing LSF expressions even in comparison to BSF was unexpected and seems paradoxical, as BSF also contains LSF in addition to HSF components. It is worth noting, however, that functional deficits induced by brain damage may not be understood simply in terms of absence of a putative function that is normally mediated by the lesioned tissue [47, 57, 58]. Rather the pathological behaviour reflects the reorganisation of dynamic interactions of the region with other interconnected structures, either due to diaschisis, hyperfunctioning, strengthening or generation of aberrant fibre connections [18, 28, 52, 59, 60]. It is therefore possible that dysfunctional cortical components are not simply “silent” when engaged by the presence of HSF presented either in isolation or in unaltered images but affect the compensatory processed carried out by subcortical structures on LSF. This issue clearly deserves further investigation in future studies.

So far, the impact of special frequency manipulation has been studied primarily in relation to affective stimuli. There is however interesting evidence showing that other aspects of face processing, besides emotion perception, may draw selectively on LSF components [19, 61]. Whether this applies also to TBI patients and to different information conveyed by faces, such as gender or age and familiarity, remains to be examined. The extension of our paradigm to other emotions, such as anger, surprise, or more social emotions like arrogance or contempt, would be helpful to detect differences in participants' performance, depending on the type of the stimulus presented. Furthermore, it would be interesting to examine how deficits and sparing of emotion perception in experimental tasks relate to every-day psychosocial functioning of TBI patients and recovery potential. By focusing on relatively preserved emotion perception in LSF, we hope to find a useful line of investigation to better define preserved functions on which rehabilitation intervention can capitalize.

## Figures and Tables

**Table 1:** Means (SEM) of the demographic and clinical characteristics of the Traumatic Brain Injured (TBI) subjects and the Healthy Controls (HC). T test for independent samples was used for group comparisons of age and education, while chi-square test was used for comparing sex distribution.

	TBI participants	HC participants	Statistics	p
	(N=14)	(N=20)		
Sex (M/F)	4 F	7 F	chi-square = 0.16	0.69

Age	38.3 (9.6)	37.7 (10.6)	t = 0.34	0.854

Education	9.8 (3.7)	9.9 (4.1)	t = 0.14	0.905

GCS	3.5 (2.9)	-	-	-

Year after onset	8.73 (4.45)	-	-	-

**Table 2:** Results of the FEEST test in the TBI group.

**FEEST**	**Score**	**Normative Mean**	**Cut-Off**		**Score**	**Normative Mean**	**Cut-Off**
**P1**				**P8**			

*Total Score*	42*∗*	51.43	45	*Total Score*	43*∗*	51.20	43
*Happy*	10	9.90	9	*Happy*	8	9.84	9
*Sad*	8	8.59	6	*Sad*	9	8.53	6
*Fear*	7	7.82	4	*Fear*	9	7.23	4
*Surprise*	4	8.54	6	*Surprise*	5	8.61	6
*Anger*	8	8.21	5	*Anger*	8	8.17	5
*Disgust*	5	8.38	6	*Disgust*	4	8.77	6

**P2**				**P9**			

*Total Score*	44*∗*	51.43	45	*Total Score*	42*∗*	51.43	45
*Happy*	8	9.90	9	*Happy*	10	9.90	9
*Sad*	9	8.59	6	*Sad*	8	8.59	6
*Fear*	7	7.82	4	*Fear*	6	7.82	4
*Surprise*	6	8.54	6	*Surprise*	3	8.54	6
*Anger*	5	8.21	5	*Anger*	8	8.21	5
*Disgust*	9	8.38	6	*Disgust*	7	8.38	6

**P3**				**P10**			

*Total Score*	43*∗*	51.20	43	*Total Score*	35*∗*	51.20	43
*Happy*	10	9.84	9	*Happy*	5	9.84	9
*Sad*	6	8.53	6	*Sad*	8	8.53	6
*Fear*	7	7.23	4	*Fear*	5	7.23	4
*Surprise*	4	8.61	6	*Surprise*	9	8.61	6
*Anger*	9	8.17	5	*Anger*	3	8.17	5
*Disgust*	7	8.77	6	*Disgust*	5	8.77	6

**P4**				**P11**			

*Total Score*	44*∗*	51.43	45	*Total Score*	40*∗*	51.20	43
*Happy*	10	9.90	9	*Happy*	10	9.84	9
*Sad*	9	8.59	6	*Sad*	10	8.53	6
*Fear*	8	7.82	4	*Fear*	10	7.23	4
*Surprise*	5	8.54	6	*Surprise*	2	8.61	6
*Anger*	7	8.21	5	*Anger*	7	8.17	5
*Disgust*	5	8.38	6	*Disgust*	1	8.77	6

**P5**				**P12**			

*Total Score*	40*∗*	51.43	45	*Total Score*	28*∗*	51.43	45
*Happy*	9	9.90	9	*Happy*	10	9.90	9
*Sad*	10	8.59	6	*Sad*	4	8.59	6
*Fear*	8	7.82	4	*Fear*	8	7.82	4
*Surprise*	3	8.54	6	*Surprise*	4	8.54	6
*Anger*	9	8.21	5	*Anger*	1	8.21	5
*Disgust*	1	8.38	6	*Disgust*	1	8.38	6

**P6**				**P13**			

*Total Score*	34*∗*	51.43	45	*Total Score*	40*∗*	51.20	43
*Happy*	10	9.90	9	*Happy*	3	9.84	9
*Sad*	8	8.59	6	*Sad*	2	8.53	6
*Fear*	2	7.82	4	*Fear*	5	7.23	4
*Surprise*	9	8.54	6	*Surprise*	3	8.61	6
*Anger*	1	8.21	5	*Anger*	6	8.17	5
*Disgust*	4	8.38	6	*Disgust*	1	8.77	6

**P7**				**P14**			

*Total Score*	27*∗*	51.43	45	*Total Score*	38*∗*	51.20	43
*Happy*	0	9.90	9	*Happy*	10	9.84	9
*Sad*	4	8.59	6	*Sad*	10	8.53	6
*Fear*	10	7.82	4	*Fear*	6	7.23	4
*Surprise*	2	8.54	6	*Surprise*	4	8.61	6
*Anger*	7	8.21	5	*Anger*	2	8.17	5
*Disgust*	4	8.38	6	*Disgust*	6	8.77	6

## Data Availability

The data used to support the findings of this study are available from the corresponding author upon request.
